# Supplementary material for: The Rise of Hypothesis-Driven Artificial Intelligence in Oncology
Source: Cancers (Basel). 2024 Feb 18;16(4):822. doi: 10.3390/cancers16040822 (PMC10886811; doi:10.3390/cancers16040822)
Supplement: Supplementary file 1 [file cancers-16-00822-s001.zip › cancers-2796700-supplementary.pdf]

Supplementary Table S1: Summary of different hypothesis-driven AI algorithms and their application in oncology research.

AI Model	AI Method	Hypothesis	Application Area	Key Advancements	Future Challenges
OncoNPC	Deep Learning	Accurate primary cancer-type predictions using OncoNPC lead to meaningful clinical benefits for patients with Cancer of Unknown Primary (CUP)	Tumor Detection	Trained on targeted NGS data to identify specific cancer types; Improved outcomes for CUP patients with concordant treatments	More diverse patient populations to show the generalizability of the AI are needed
MALANI	Support vector machine (SVM)	Class II cancer genes act as "signal linkers" between mutated and differentially expressed genes; A machine learning-based approach like MALANI can identify these elusive Class II cancer genes	Cancer Genome	Focuses on identifying "dark" cancer genes; Uses SVM to detect dark genes or Class II genes	Experimental validation of identified genes and expansion to more cancer types and larger sample sizes for robustness can be done
Symbolic Regression	Genetic Algorithm	Incorporating three filters into GP-based SR, along with nonlinear least-squares optimization, improves predictive ability of SR models while generating simpler and more interpretable expressions	Drug Response	Generates interpretable QSAR/QSPR models; Bridges gap between complex biological relationships and therapeutic interventions	Refining approach and generating simple predictive expressions for all types of analysis

AI Model	AI Method	Hypothesis	Application Area	Key Advancements	Future Challenges
Biologically Informed Deep Neural Network (P-Net)	Deep Learning	Evolutionarily conserved biological interactions and hierarchical structures hold critical information flows; Deep neural networks can recapitulate these interactions and shed light on underlying cancer biology	Cancer State Determination	Biologically informed deep learning model based on sparse deep learning architectures; Integrates diverse molecular features for enhanced predictability	Current model's performance is contingent on the quality and accuracy of biological pathway annotations, which can be further improved; Validation of model for other cancer types except for prostate cancer can be done
ANNE (Weight Engineering)	Artificial Neural Network	Weight engineering approach decodes gene-gene associations from ANNs trained on omics data; Provides mechanistic insights into clinical phenotypes	Gene-Gene Association	Focuses on extracting biologically meaningful knowledge from ANNs; Uses weight engineering to interpret network connections	Findings would benefit from further experimental validation and application to other cancer types
Spatially Informed AI for Cell Organization (SPIN-AI)	Deep Learning	Spatially predictive genes (SPGs) govern cellular organization; Deep learning on spatial transcriptomic data can uncover spatial relationships in cellular environments	Tumor Microenvironment	Utilizes deep learning to predict spatial coordinates of gene expression spots; Provides insights into cellular organization in pathological microenvironments	Current model's effectiveness may be influenced by specific characteristics of tumor microenvironment; Applicability to other biological conditions or tissues can be explored

AI Model	AI Method	Hypothesis	Application Area	Key Advancements	Future Challenges
Visible Machine Learning approach (DCell)	Deep Learning	Hierarchical structure of biological systems encodes valuable information; Incorporating extensive knowledge of cell biology makes DCell a visible neural network with an interpretable inner structure	Phenotype Prediction	Models eukaryotic cell's hierarchical structure and function; Accurately simulates cellular growth influenced by genetic interactions	Effectiveness of the current model is influenced by specific characteristics of the cellular system; Applicability to other organisms or cell types requires further validation
